# Evaluation of the Neurogenic Potential in the Rat Inferior Colliculus from Early Postnatal Days Until Adulthood

**DOI:** 10.1007/s12035-020-02151-6

**Published:** 2020-10-03

**Authors:** Jonas Engert, Kristen Rak, Linda Bieniussa, Miriam Scholl, Rudolf Hagen, Johannes Voelker

**Affiliations:** Department of Oto-Rhino-Laryngology, Plastic, Aesthetic and Reconstructive Head and Neck Surgery, Comprehensive Hearing Center, Universitaetsklinikum Wuerzburg, Josef-Schneider-Strasse 11, D-97080 Wuerzburg, Germany

**Keywords:** Central auditory pathway, Inferior colliculus, Neural progenitor cells, Neural stem cells, Neurosphere

## Abstract

Neural stem cells (NSCs) have been recently identified in the inferior colliculus (IC). These cells are of particular interest, as no casual therapeutic options for impaired neural structures exist. This research project aims to evaluate the neurogenic potential in the rat IC from early postnatal days until adulthood. The IC of rats from postnatal day 6 up to 48 was examined by neurosphere assays and histological sections. In free-floating IC cell cultures, neurospheres formed from animals from early postnatal to adulthood. The amount of generated neurospheres decreased in older ages and increased with the number of cell line passages. Cells in the neurospheres and the histological sections stained positively with NSC markers (Doublecortin, Sox-2, Musashi-1, Nestin, and Atoh1). Dissociated single cells from the neurospheres differentiated and were stained positively for the neural lineage markers β-III-tubulin, glial fibrillary acidic protein, and myelin basic protein. In addition, NSC markers (Doublecortin, Sox-2, CDK5R1, and Ascl-1) were investigated by qRT-PCR. In conclusion, a neurogenic potential in the rat IC was detected and evaluated from early postnatal days until adulthood. The identification of NSCs in the rat IC and their age-specific characteristics contribute to a better understanding of the development and the plasticity of the auditory pathway and might be activated for therapeutic use.

## Introduction

Neurogenesis is the formation of new nerve cells from precursor and stem cells, which takes place both during embryogenesis and in some regions of the adult nervous system. Following the discovery of adult neurogenesis in the subventricular zone [1] and the dentate gyrus of the hippocampus [2], these brain regions were intensively investigated regarding their neurogenic potential. However, the existence of neural stem cells (NSC) has also been demonstrated in further parts of the mammalian central nervous system, such as the cortex [3], the striatum [4], the septum [4], the spinal cord [5], the dorsal vagal complex [6], and the optic nerve [3]. The main characteristics of NSCs are their capacity of mitotic self-renewal and their potential to differentiate into neural progenitor cells and all cells of the neuroectodermal lineage, including neurons, astrocytes, and oligodendrocytes [7].

Furthermore, NSCs have been identified in the inner ear and the auditory pathway. NSCs were initially described in the utricle of the vestibular organ [8]. Since then, a neurogenic potential has been confirmed in the spiral ganglion [9], the lesser epithelial ridge [10], the organ of Corti, as well as the stria vascularis [11]. Furthermore, neurogenic niches were found in the centrally located parts of the auditory pathway. A stem cell potential of the cochlear nucleus (CN) was found in both rats [12] and mice [13].

The existence of NSCs was also shown in the inferior colliculus (IC) of young postnatal mice [14] and rats [15]. These cells have the ability of mitotic self-renewal, which was demonstrated by their capacity to form neurospheres over several passages and by BrdU assays. Also, it has been demonstrated that these cells can differentiate into progenitor cells and all cell types of the neuroectodermal cell line, including neurons, astrocytes, and oligodendrocytes. The IC is the fourth relay station of the ascending auditory pathway and is located in the midbrain, where, together with the superior colliculi, it forms the lamina quadrigemina. The IC processes ascending information from the superior olivary complex as well as from the ipsilateral and contralateral CN [16]. Its efferent axons lead to the medial geniculate body and the primary auditory cortex.

Hearing loss causes functional changes in the IC. These changes show age-related characteristics and can also be detected in adult animals. Segmental cochlear lesions cause changes in the central nucleus of the IC. As a result, lesion-associated frequencies are no longer represented on the tonotopic map, and adjacent frequencies are increasingly mapped. The lesion-induced tonotopic reorganization is more pronounced in postnatal than in adult animals [17]. Furthermore, changes in continuous amplitude response characteristics of post-synaptic field potentials after acute acoustic hyperstimulation were detected on the surface of the IC of adult rats [18]. Not much is yet known about the anatomical basis of the functional reorganization of the IC after cochlear lesions. However, some anatomical changes in the central auditory pathway after acoustic deprivation have been described, including transsynaptic degeneration [19] and reduction of the size of neurons [20]. Furthermore, investigations of auditory brainstem connections showed that age-related differences in the formation of projecting neurons occur after cochlear removal [21].

Since NSCs have been discovered in the IC of postnatal rats, the question of age-related changes in the neurogenic potential of IC arises. Therefore, the neurosphere forming capacity, the presence of NSC markers in neurospheres and tissue sections, as well as the ability to differentiate was examined in the different age groups of animals up to postnatal day 48.

## Materials and Methods

### Animal Dissection

Postnatal day (PND) 6, 12, 24, and 48 Sprague-Dawley rats (Charles River®) were sacrificed by cervical dislocation. The skull was opened midsagittally, and its bony parts were removed. After the cranial nerves were dissected, the brain, including the brainstem, was removed from the skull base and transferred into Neurobasal® medium (Thermo-Fisher Scientific®) at room temperature. Using a stereomicroscope (OPMI1, Zeiss®), the cerebrum and brainstem were separated by a coronary incision above the lamina tecti, and the brainstem was freed from meningeal tissue and blood vessels. After identifying the IC, it was removed by blunt preparation. The isolated tissues were transferred either into sterile DPBS solution or into Neurobasal® medium at room temperature for further processing. All procedures were performed under antiseptic conditions. All experiments were performed in accordance with the guidelines for animal experimentation under German law. Since only organ removal was performed in sacrificed animals, there was no need to obtain consent from the local animal committee (German Animal Protection Act).

### Neurosphere Assay and Passaging

Accutase (PAA Laboratories®) was used to dissociate the neuronal tissue of IC enzymatically in a ThermoMixer (Eppendorf®) at 37 ° C and 500 rpm. Every 10 min, the suspension was triturated until visible portions of the tissue were not detectable anymore. The solution was centrifuged at 1000 rpm for 5 min (Centrifuge 5810, Eppendorf®) and the pellet suspended in neural stem cell medium, containing Neurobasal® medium (Thermo-Fisher Scientific®), 1% GlutaMAX® supplement, 2% B27® supplement without retinoic acid and 1% penicillin/streptomycin (Invitrogen®). EGF and FGF-2 (PeproTech®) were added to the cultures at a final concentration of 10 ng/ml each. This medium will be referred to as “NSC-medium.” Before culturing the cells, a mixture of a sample from the suspension and trypan blue (Invitrogen®) was used to count the number of viable cells in an improved Neubauer hemocytometer (ZK06, Hartenstein®). All cells dissociated from both IC of one animal were cultured in hydrophobic cell culture flasks (CellStar, filter-top, 25 cm^2^, Greiner® Bio One) at 37 °C and 5% CO_2_. Initially, a suspension was carried out in 4 ml of NSC medium, and 2 ml of freshly prepared NSC medium was added every 4 days. The number of primary cell spheres was determined after 30 days in culture with an inverted microscope (Leica® DMI 4000B and DMI-8) in transmitted light technique and recalculated to 100,000 cultured cells per IC.

The dissociation of the neurospheres was performed mechanically in order to passage the cells. After the dissociation, the single cells were centrifuged for 5 min at 1000 rpm, and the pellet was rinsed with PBS solution (Gibco®). Subsequently, PBS was removed, and the single cells were resuspended in fresh NSC medium and cultivated at 37 °C/5% CO_2_ for 30 days in 25-cm^2^ filter top cell culture flasks (CellStar, Greiner® bio-one). The absolute number of viable cells was determined between each passaging step.

### Plating of Spheres

Neurospheres were taken out of the cell cultures and plated on glass coverslips coated with Poly-D-lysine (100 μg/ml, Serva Electrophoresis®) and Laminin (10 μg/ml, BD Biosciences®). The plated neurospheres were cultivated in NSC medium for 24 h for further analysis at 37 ° C/5% CO_2_.

### Plating of Single Cells and Cell Differentiation

The suspension from the propagated cell cultures was centrifuged (1000 rpm, 5 min), and the cell pellet washed once with DPBS. After removing DPBS, Accutase® was added to dissociate the cells enzymatically. The suspension was incubated for 15 min at 37 ° C and 500 rpm in a ThermoMixer (Eppendorf®) and triturated every 5 min. After centrifugation and suction of Accutase®, the pellet was resuspended in the NSC medium in order to plate single cells for differentiation experiments. In 4-well dishes (Greiner® bio one), the single cells were plated on glass coverslips coated with Poly-D-lysine (100 μg/ml, Serva Electrophoresis®) and Laminin (10 μg/ml, BD Biosciences®) and cultivated in NSC medium. After 24 hours, a differentiation medium (DIF medium) was added, composed of Neurobasal®, GlutaMAX®, and B27 with retinoic acid. The single cells were plated at a density of 100 cells/mm^2^ (8000 cells/coverslip) and cultivated for 5 days at 37 °C and 5% CO_2_.

### Fixation and Immunocytochemistry

At the end of the experiment, the plated cells or neurospheres on the coverslips were fixed with 4% paraformaldehyde (PFA, Sigma-Aldrich®) for 30 min and with acetone for 5 min. For blocking, a solution of 10% bovine serum albumin (BSA, Sigma-Aldrich®) in TBS-T buffer solution (200 mM Tris-base (pH = 8), 8% NaCl, 1% Tween-20, Sigma-Aldrich®) was used. The cells were incubated with the following primary antibodies at 5 °C overnight in 1% BSA solution and TBS-T: rabbit polyclonal against Doublecortin (DCX) (1:1000, Abcam®), rabbit polyclonal against Sox-2 (1:100, Abcam®), rabbit polyclonal against Musashi-1 (1:100, Abcam®), rabbit polyclonal against Atoh1 (1:100, Santa Cruz®), mouse monoclonal against Nestin (1:500, Millipore®), mouse monoclonal against β-tubulin (1:500; Sigma-Aldrich®), rabbit polyclonal against β-tubulin (1:50, Santa Cruz®), mouse monoclonal against β-III-tubulin (1:500; Abcam®), rabbit polyclonal against β-III-tubulin (1:500; Abcam®), mouse monoclonal against glial fibrillary acidic protein (GFAP) (1:500; Millipore®) or rabbit polyclonal against myelin basic protein (MBP) (1:100, Sigma-Aldrich®). After being rinsed three times with TBS-T, the cells were incubated for 1 h in a 1% BSA solution at room temperature with goat anti-rabbit or goat anti-mouse secondary antibody coupled to Alexa 488 or Alexa 555 (1:800, 1.500, Invitrogen®) and 5 μg/ml DAPI (1:5000, Sigma-Aldrich®). The cells were then washed three times with TBS-T, and the coverslips were embedded in Moviol (Sigma-Aldrich®).

### Histological Sectioning and Immunohistochemistry

After dissection, the IC was fixed in 4% paraformaldehyde (Sigma-Aldrich®) for 1 h and incubated in three steps with 10, 20, and 30% saccharose in ascending order for 24 h each. Tissue was then cryoprotected in Tissue-Tek O.C.T. (Sakura®) and frozen in liquid nitrogen. Using the cryostat (CM1510S, Leica®), preparations were cut in sections of 9 μm, mounted on superfrost slides (Hartenstein®) and stored for immunofluorescence analysis. Therefore, sections were post-fixed in 4% PFA for 5 min, washed three times in TBS-T and blocked in 10% BSA in 0.3% Triton X-100 (Sigma-Aldrich®) for 1 h. For immunohistochemistry, the sections were incubated with the following primary antibodies in 1% BSA solution in 0.3% Triton X-100 for 24 h: mouse monoclonal against Nestin (1:500, Millipore®), mouse monoclonal against β-tubulin (1:500, Sigma Aldrich®), rabbit polyclonal against β-III-tubulin (1:500, Abcam®), rabbit polyclonal against Atoh1 (1:100, Santa Cruz®), rabbit polyclonal against Musashi-1 (1:100, Abcam®), or rabbit polyclonal against Sox-2 (1:100, Abcam®). After three washing steps with TBS-T, the sections were incubated with the secondary antibodies, coupled to Alexa 488 or Alexa 555 (1:800, 1:500, Invitrogen®) and 5 μg/ml DAPI (1:5000, Sigma-Aldrich®). The specimens were then rinsed three times with TBS-T and embedded in Moviol (Sigma-Aldrich®).

### RNA Extraction and First-Strand cDNA Synthesis

After the dissection, the IC samples from rats of different ages (*n* = 4 animals/age group) were used for a qRT-PCR array. First, total RNA was isolated according to the RNeasy Micro Kit (Qiagen®). The RNA of the different samples of one age group was pooled. The RNA concentration (A260) and quality (A260/A280 ratio) were determined with a NanoDrop spectrophotometer (Thermo Scientific®). For the A260/A280 ratio, samples in the range of 2.0 ± 0.2 were accepted. To obtain cDNA, 500 ng of RNA per age group was synthesized into cDNA using the RT2 First Strand Kit (Qiagen®) according to the instructions for use.

### Rat Neurogenesis RT2 Profiler^TM^ PCR Array

Of the obtained cDNA, 102 μl were labeled with 1350 μl RT2 SYBR Green Mastermix (Qiagen®) and 1248 μl dH2O. From this mixture, 25 μl per well were transferred to a 96-well PCR array plate containing the genes DCX, Sox-2, CDK5R1, and Ascl-1 (Rat Neurogenesis RT2 Profiler^TM^ PCR Array, Qiagen®). Additionally, five household genes (Actb, B2m, Hprt1, Ldha, Rplp1) and three quality controls for RNA contamination and PCR quality were on each plate. Three PCR array plates were evaluated per age group. The PCR reactions were performed with a Step One Plus (Applied Biosystems®) and according to the following cycling program: the plates were first incubated for 10 min. at 95 °C and then subjected to 40 cycles, each consisting of two phases. The plates were incubated for 15 s at 95 °C and then incubated for 1 min at 60 °C.

### Digital Pictures, Quantification, and Statistical Analyses

Digital images of the cell cultures and preparations were taken with a Leica® DMI8 fluorescence microscope and Leica Application Suite X software v3.0.1, Leica®. To quantify the number of neurospheres, all culture flasks were scanned using the transmitted light technique in tile scan mode. The digital images of microscopic images were exported directly from the Leica Application Suite software in TIFF format. The final image composition was done with the Adobe® InDesign CC 2020 v15.0.2 software. Tissue sections were analyzed with an Olympus® Fluoview FV3000 confocal laser scanning microscope and exported with the Fiji/ImageJ V2.0.0 software [22]. All collected data was compiled using the Microsoft® Excel 2018 V16.19 spreadsheets and statistically analyzed with the GraphPad® Prism 7.0a software. The Shapiro-Wilk normality test was used to determine whether a Gaussian normal distribution was present. For further analysis, a one-way ANOVA with a post-hoc Tukey’s multiple comparison test was used in case of normal distribution. A *p* value less than 0.05 was considered significant. If the data set followed a Gaussian normal distribution, mean and standard error of the mean (SEM) are displayed, whereas without Gaussian normal distribution, mean and standard deviation (SD) are depicted.

The analysis of the gene expression of the individual factors was performed according to the instructions of the Gene Globe Data Analysis Center (Qiagen®). Due to the inversely proportional relationship between the threshold cycle (CT) and the pure expression level of a gene, and the doubling of the amount of a gene product per amplification cycle, the expression level for each gene was determined as ^2-CT^. To normalize the expression level of a gene of interest (GOI), it was related to the expression of the mean of the HKGs: 2^-CT(GOI)/2-CT(HKG)^ = 2^-[CT(GOI)-CT(HKG)]^ = 2^-∆CT^. The resulting 2^-∆CT^ values were compared between different age groups using a two-stage, unpaired Student’s *t* test.

## Results

### Neurosphere-Forming Capacity and Single-Cell Count

**Fig. 1:**
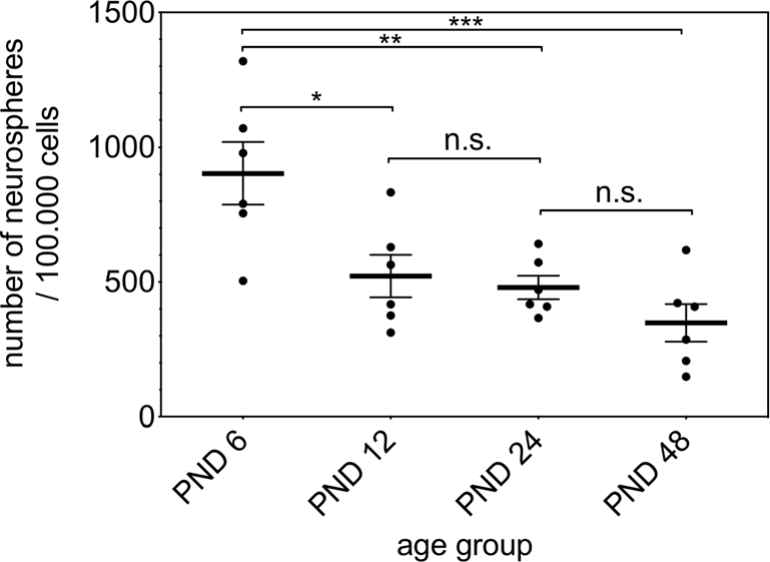
The ratio of the number of primary neurospheres to 100,000 seeded vital cells of the rat IC shows an age-dependent decrease. The highest neurosphere-forming capacity was found at PND 6 and the lowest at PND 48. The central horizontal bars show the mean; error bars depict the standard error of the mean; dots each represent one single cell culture, *n* = 6; asterisks indicate the level of significance, *p < 0.05, **p < 0.005, ***p < 0.001, ****p < 0.0001

The formation of primary neurospheres was detected in cell cultures of the dissociated cells from the IC. For estimation, a ratio of primary neurospheres per 100,000 vital cells was calculated (PND 6903 ± 282.4, PND 12 522 ± 193.3, PND 24 480 ± 106.6, PND 48 348 ± 170.7) (*n* = 6). This evaluation showed a significant decrease in the ratio between PND 6 and the following age groups (PND 6 vs. PND 12, *p* = 0.0164; PND 6 vs. PND 24, *p* = 0.0073; PND 6 vs. PND 48, *p* = 0.0005). No significant difference was found between PND 12 and PND 24 (*p* = 0.9825), and PND 24 and PND 48 (*p* = 0.4477) (Fig. 1).

**Fig. 2:**
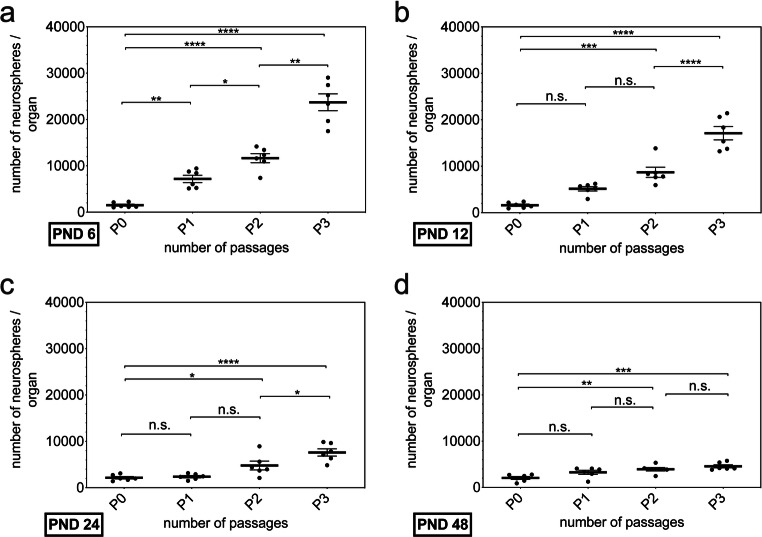
The number of neurospheres per organ formed increased with the rising number of passages from PND 6 to PND 48. **a**–**d** All examined age groups showed a significant increase from P0 to P3. **a** Within the age group, the number of neurospheres constantly increased significantly at PND 6. **b** and **c** At PND 12 and PND 24, the increase between P2 and P3 was significant. **d** No significant increase between adjacent passages was observed at PND 48. The central horizontal bars show the mean; error bars depict the standard error of the mean; dots each represent a cell culture, ***n*** = 6; asterisks indicate the level of significance, *p < 0.05, **p < 0.005, ***p < 0.001, ****p < 0.0001

In addition, the formation of secondary, tertiary, and quaternary neurospheres from PND 6 throughout PND 48 was analyzed. Therefore, the amount of primary neurospheres per animal was analyzed (*n* = 6) (PND 6 1505 ± 527.8, PND 12 1606 ± 583.2, PND 24 2152 ± 636.4, PND 48 2045 ± 753.3) (Fig. 2). With increased passages, the number of neurospheres increased within all examined age cohorts (P0 vs. P3) (PND 6, *p* < 0.0001; PND 12, *p* < 0.0001; PND 24, *p* < 0.0001; PND 48, *p* < 0.001). The capacity to generate neurospheres varied between age cohorts and passage numbers (Fig. 2). The increases between the various passages at PND 6 were all significant (P0 vs. P1, *p* = 0.0088; P1 vs. P2, *p* = 0.0449; P2 vs. P3, *p* < 0.0001) (Fig. 2). At PND 12 and PND 24, no significant changes between primary culture and passage 1 (PND 12, *p* = 0.0664; PND 24, *p* = 0.9947) and between passage 1 and passage 2 (PND 12, *p* = 0.0663; PND 24, *p* < 0.0667) (Fig. 2) were found. However, between passage 2 and passage 3, significant increases were detected at both age stages (PND 12, *p* < 0.0001; PND 24, *p* = 0.0265) (Fig. 2). The increase between a passage and its immediately adjacent passage showed no significance at PND 48 (P0 vs. P1, *p* = 0.12; P1 vs. P2, *p* = 0.5729; P2 vs. P3, *p* = 0.6346) (Fig. 2).

**Fig. 3:**
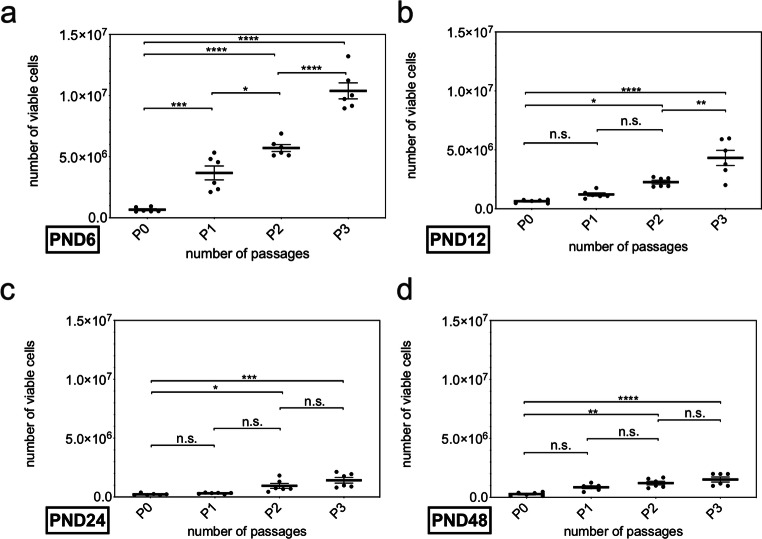
The number of viable cells within the neurospheres increased with the rising number of passages from PND 6 to PND 48. **a**–**d** All examined age groups showed a significant increase from P0 to P3. **a** Within the age group, the number of vital cells within the neurospheres constantly increased significantly at PND 6. **b** At PND 12, the increase between P2 and P3 was significant. **c** and **d** No significant increase between adjacent passages was observed at PND 24 and PND 48. The central horizontal bars show the mean; error bars depict the standard error of the mean; dots each represent a cell culture, ***n*** = 6; asterisks indicate the level of significance, *p < 0.05, **p < 0.005, ***p < 0.001, ****p < 0.0001

Additionally, the number of viable cells in primary cultures was determined (*n* = 6) (PND 6 661,833 ± 187,318; PND 12 638,333 ± 140,487; PND 24 225,667 ± 82,902; PND 48 276,833 ± 152,053) (Fig. 3). The number of viable cells increased with higher passage numbers within all examined age groups (P0 vs. P3) (PND 6, *p* < 0.0001; PND 12, *p* < 0.0001; PND 24 *p* < 0.001; PND 48, *p* < 0.0001) (Fig. 3). At PND 6, a significant increase was detected between all examined passages (P0 vs. P1, *p* < 0.001; P1 vs. P2, *p* = 0.0236; P2 vs. P3, *p* < 0.0001) (Fig. 3). Changes between primary culture and passage 1 (*p* = 0.6329) and passage 1 and passage 2 (*p* = 0.1592) showed no significance at PND 12, whereas the increase between passage 2 and passage 3 was significant (*p* = 0.0018) (Fig. 3). At PND 24 and PND 48, no significant change was detected between adjoined passages (PND 24 P0 vs. P1, *p* = 0.9811; P1 vs. P2. *p* = 0.0517; P2 vs. P3, *p* = 0.2013; PND 48 P0 vs. P1, *p* = 0.0503; P1 vs. P2, *p* = 0.3415; P2 vs. P3, *p* < 0.4766) (Fig. 3).

### Identification and Quantification of NSC Markers in Neurospheres

**Fig. 4:**
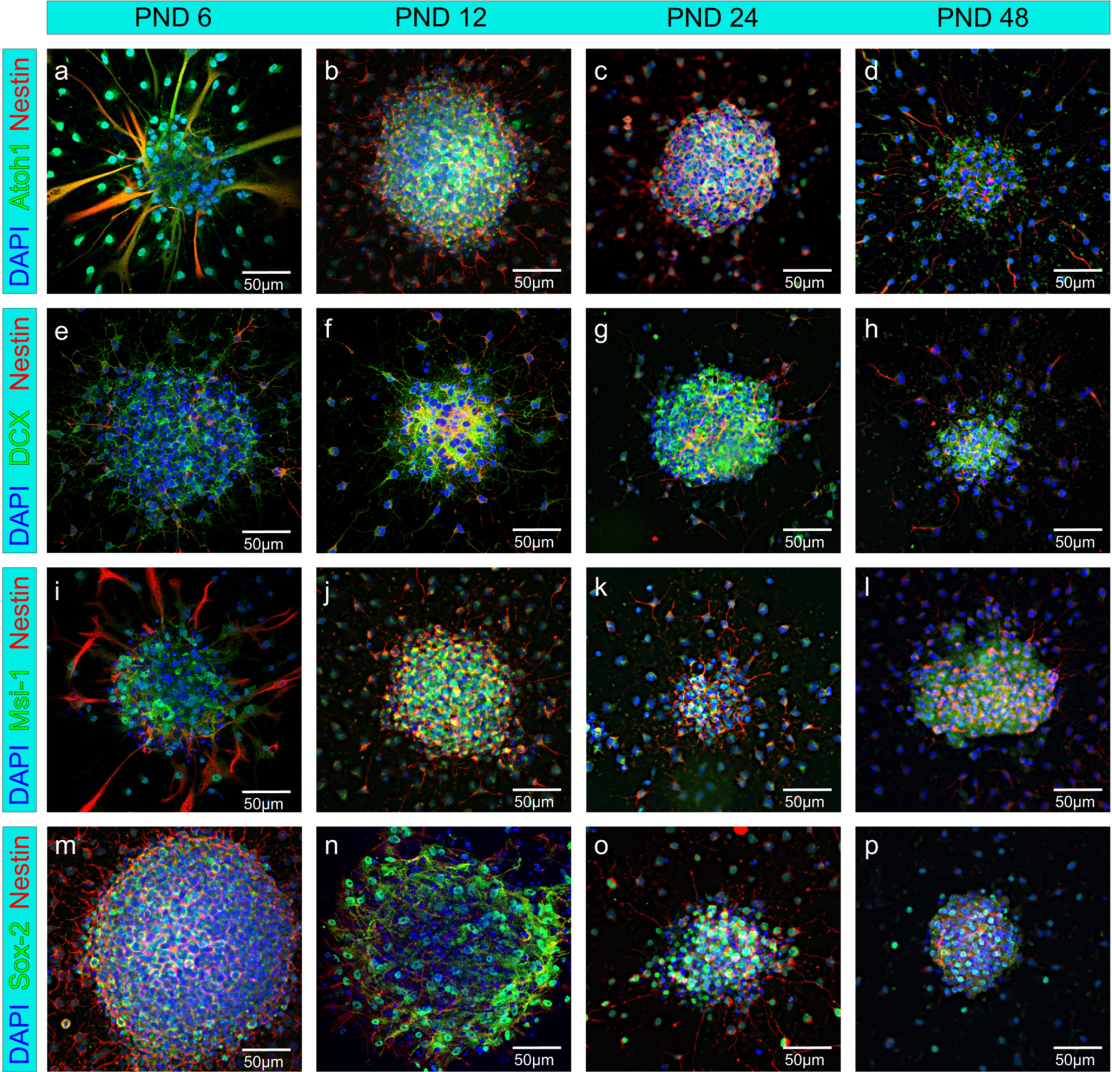
Neural stem cell markers are expressed in neurospheres from progenitor cells of the rat IC from PND 6, PND 12, PND 24, and PND 48. (a–p) Cells inside the neurospheres and cells emigrating from the spheres were stained positive for the neural progenitor marker Nestin (red), cell nuclei were stained blue by DAPI. (a–d) The nucleus of cells inside the sphere and of its branches showed positive labeling for transcription factor Atoh1 (green). (e–h) The cytoplasm of emigrating cells and cells inside the neurospheres were stained by neural migration protein DCX (green). (i–l) The neural stem cell marker Musashi-1 (green) was positive in the cytoplasm of cells inside the neurosphere and its branches. (m–p) The nucleus of cells within the neurospheres and its branches showed positive labeling for the transcription factor Sox-2 (green)

The neurospheres were stained with the markers DCX, Sox-2, Musashi-1, Atoh1, and Nestin. The expression of the transcription factors Atoh1 (Fig. 4a–d) and Sox-2 (Fig. 4m–p) was found in all ages and few cells showed a colocalization with DAPI in the nuclei of the cells. Neuronal migration maker DCX (Fig. 4e, f) and RNA-binding protein Musashi-1 (Fig. 4i–l) were detected within the cytoplasm of the sphere-forming cells at PND 6, PND 12, PND 24, and PND 48. In addition, the cells within the neurospheres of all age groups showed positive labeling of the progenitor marker Nestin (Fig. 4a–p) in their cytoplasm.

**Fig. 5:**
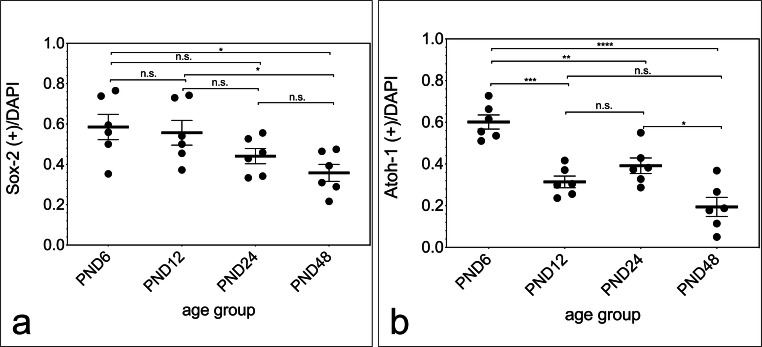
Age-dependent evaluation of neural stem cell marker–positive cells in relation to DAPI-positive cells in neurospheres from the rat IC. **a** The relative expression of the transcription factor Sox-2 showed a significant decrease between PND 6 and PND 48, as well as PND 12 and PND 48. **b** With the exception between PND 12 and PND 24, and PND 12 and PND 48, the relative expression of the bHLH transcription factor Atoh1 showed a significant decrease with increasing age. The central horizontal bars show the mean; error bars depict the standard error of the mean; dots each represent a cell culture, ***n*** = 6; asterisks indicate the level of significance: *p < 0.05, **p < 0.005, ***p < 0.001, ****p < 0.0001

The analysis of marker-positive cells in relation to DAPI-positive cells in the neurospheres of all age groups investigated was performed (Fig. 5). The ratio of Sox-2-positive to DAPI-positive cells showed a significant decrease between the age groups PND 6 (0.5850 ± 0.1535) and PND 48, as well as PND 12 (0.5565 ± 0.1498) and PND 48 (0.3577 ± 0.1032) (PND 6 vs. PND 48, *p* = 0.0161; PND 12 vs. PND 48, *p* = 0.0374). The comparison of the other age groups showed no significant changes (PND 6 vs. PND 12, *p* = 0.9804; PND 6 vs. PND 24, *p* = 0.2566; PND 12 vs. PND 24, *p* = 0.3251; PND 24 vs. PND 48, *p* = 0.5971) (Fig. 5a). The evaluation concerning Atoh1 showed a significant decrease between PND 6 (0.6013 ± 0.08311) and all other age groups (PND 6 vs. PND 12, *p* = 0.0005, PND 6 vs. PND 24, *p* = 0.0078, PND 6 vs. PND 48, *p* < 0.0001), and between PND 24 (0.3916 ± 0.09146) and PND 48 (0.1940 ± 0.1123) (PND 24 vs. PND 48, *p* = 0.0121). An exception was the comparison between PND 12 (0.3141 ± 0.06849) and PND 24 as well as PND 12 and PND 48 (PND 12 vs. PND 24, *p* = 0.5071; PND 12 and PND 48, *p* = 0.1687) (Fig. 5b).

### Capacity to Differentiate into Cells of the Neuronal Lineage

**Fig. 6:**
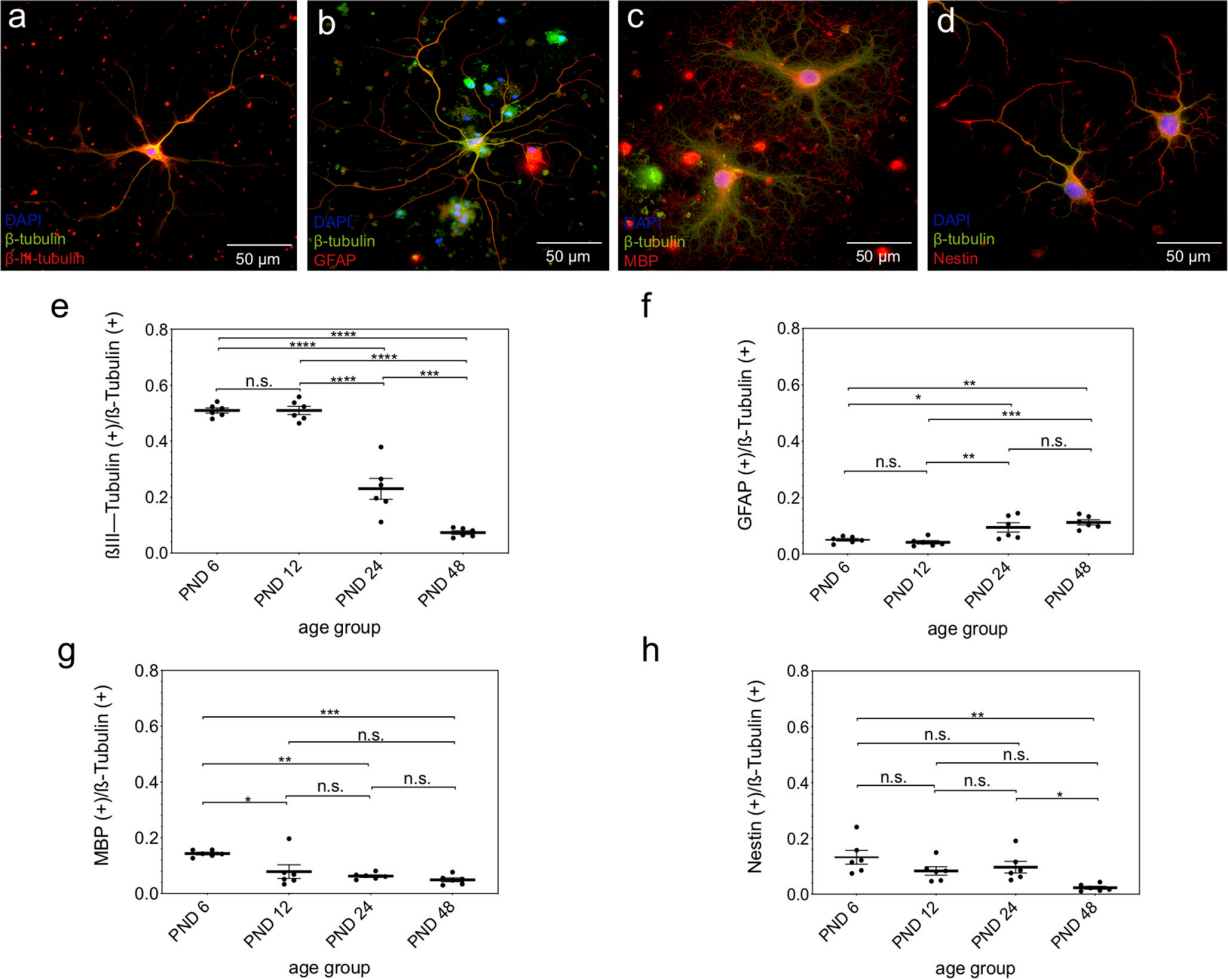
Age-dependent capacity of cells from neurospheres of the rat IC to differentiate into all cell types of the neuroectodermal cell lines. **a**–**d** Immunofluorescence images of differentiated cells from the rat IC at PND 48. The cells were incubated for 5 days on glass coverslips in differentiation medium. **a** β-III-tubulin (red) marked cells differentiated into neurons. **b** Astrocytes were stained with GFAP (red). **c** Oligodendrocytes are positively labeled by MBP (red). MBP marked the peripheral myelination processes, whereas the proximal portions are β-tubulin-positive (green). **d** Undifferentiated progenitor cells were stained with Nestin (red). The cytoskeleton of all viable cells was stained with β-tubulin (green). Cell nuclei were stained with DAPI (blue). **e**–**h** Evaluation of differentiation marker-positive cells in relation to β-tubulin-positive cells from PND 6 to PND 48. **e** The neuron-specific marker β-III-tubulin showed a constant significant decrease of expression with increasing age except between PND 6 and PND 12. **f** Between PND 6 and PND 48, with a significant increase between PND 12 and PND 24, there was a significant increase in the expression of the marker GFAP. **g** A significant age-dependent decrease between PND 6 and PND 48 of the expression of MBP was found with the most significant decrease between PND 6 and PND 12. **h** The neural progenitor marker Nestin is significantly reduced in expression with increasing age between PND 6 and PND 48. The central horizontal bars show the mean; error bars depict the standard error of the mean; dots each represent a cell culture, ***n*** = 6; asterisks indicate the level of significance: *p < 0.05, **p < 0.005, ***p < 0.001, ****p < 0.0001

Single cells from dissociated neurospheres of all examined ages developed into neurons and neuroglia. The neuron-specific marker β-III-tubulin (Fig. 6 a) was labeled positively in axons and the cytoplasm of neurons. Astrocytes were identified using GFAP (Fig. 6b). MBP (Fig. 6c) plays an important role in the myelination of nerves and therefore stained the myelin sheath of oligodendrocytes. In addition, neural precursor marker Nestin (Fig. 6d) was used to detect neural progenitor cells in early stages of development. Throughout all age cohorts, Nestin (Fig. 6h) was labeled positive in the cytoplasm of cells.

The number of marker-positive single cells was evaluated in relation to the β-tubulin-positive cells on the respective cover glass. The ratio of β-III-tubulin-positive cells to β-tubulin-positive cells showed an age-related decrease (PND 6 0.5097 ± 0.02224, PND 12 0.5098 ± 0.03613, PND 24 0.2297 ± 0.09029, PND 48 0.07332 ± 0.01564) with exception of between PND 6 and PND 12, with no significant difference (PND 6 vs. PND 12, *p* > 0.99; PND 6 vs. PND 24, *p* < 0.0001; PND 6 vs. PND 48, *p* < 0.0001; PND 12 vs. PND 24, *p* < 0.0001; PND 12 vs. PND 48, *p* < 0.0001; PND 24 vs. PND 48, *p* < 0.002) (Fig. 6e). The GFAP-specific evaluation showed a significant increase between PND 12 (0.04183 ± 0.01444) and PND 24 (*p* = 0.0074), PND 12 and PND 48 (0.1126 ± 0.02232) (*p* = 0.0004), PND 6 (0.05064 ± 0.01128) and PND 24 (*p* = 0.0285), and PND 6 and PND 48 (*p* = 0.0017). The differences in the cell ratio between PND 6 and PND 12 (*p* = 0.9263), as well as PND 24 (0.0946 ± 0.04037) and PND 48 (*p* = 0.5998) were without significance (Fig. 6f). Regarding MBP, a significant decrease between PND 6 (0.1436 ± 0.01144) and the following age groups (PND 12 0.07882 ± 0.05974, PND 24 0.06275 ± 0.01104, PND 48 0.04929 ± 0.01694) was shown (PND 6 vs. PND 12, *p* = 0.0111; PND 6 vs. PND 24, *p* = 0.0016; PND 6 vs. PND 48, *p* = 0.0003). The other differences between the ratios of the different age groups were not significant (PND 12 vs. PND 24, *p* = 0.8219; PND 12 vs. PND 48, *p* = 0.4033; PND 24 vs PND 48, *p* = 0.8849) (Fig. 6g). Analysis of the ratio between Nestin-positive and β-tubulin-positive cells showed a significant decrease between PND 6 (0.1324 ± 0.06071) and PND 48 (0.02308 ± 0.01266) (*p* = 0.0019), and PND 24 (0.09686 ± 0.05121) and PND 48 (*p* = 0.0421). The other differences between the age groups (PND 12 0.0833 ± 0.0373) were not significant (PND 6 vs. PND 12, *p* = 0.2522; PND 6 vs. PND 24, *p* = 0.5204; PND 12 vs. PND 24, *p* = 0.9509; PND 12 vs. PND 48, *p* = 0.1194) (Fig. 6h).

### Identification of NSC Markers in Histological Sections

**Fig. 7:**
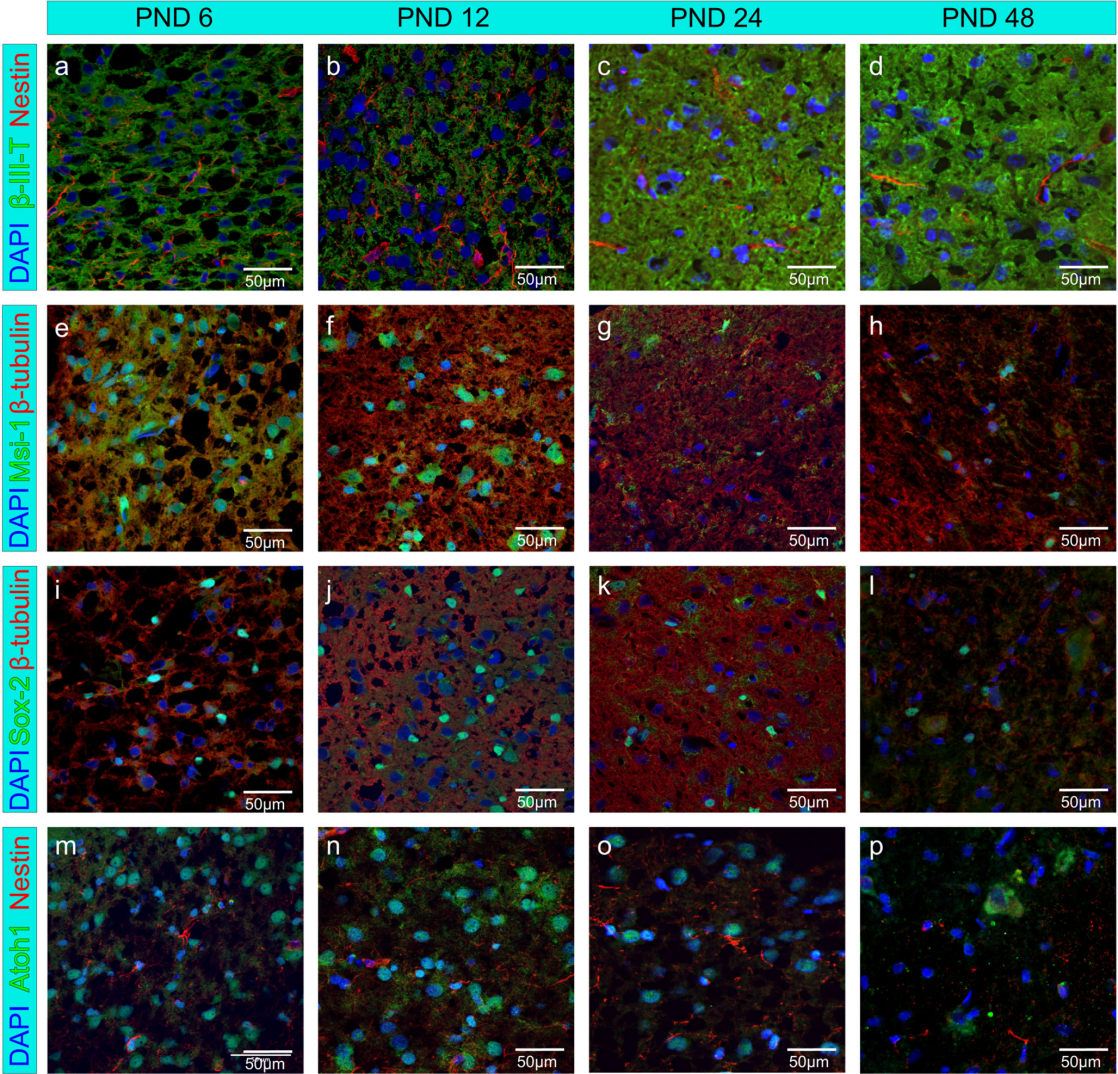
Neural progenitor and stem cell markers are expressed in histological sections of the rat IC from PND 6, 12, 24, and 48. (a–p) Cell nuclei are stained blue by DAPI. a–d The neural progenitor marker Nestin (red) and neuron-specific marker β-III-tubulin (green) are expressed in the cytoplasm of cells, but no co-labeling of both markers was detected (a as published in [15]). (e–h) The neural stem cell marker Musashi-1 (green) is expressed perinuclear. β-tubulin (red) is positive in the cytoplasm of cells. (i–l) The transcription factor Sox-2 (green) is expressed in the nucleus of cells. β-tubulin (red) is expressed in the cytoplasm of the cells. (m–p) The transcription factor Atoh1 (green) is expressed in the nucleus of cells, and the cytoplasm of cells was positive for Nestin (red)

Histological sections were examined for the presence of the markers Sox-2, Atoh1, Musashi-1, and Nestin. In sections from PND 6 to PND 48, Sox-2 (Fig. 7e–h) and Atoh1 (Fig. 7m–p) were found in the nuclei of the cells in colocalization with DAPI. The expression of Musashi-1 (Fig. 7i–l) was detected in the nuclei, collocated with DAPI, as well as in the cytoplasm of cells and was detectable in the sections of all examined ages. Throughout all age cohorts, Nestin (Fig. 7a–d) was positively labeled in the cytoplasm of cells.

### Molecular Genetic Analysis of NSC Markers Using qRT-PCR

**Fig. 8:**
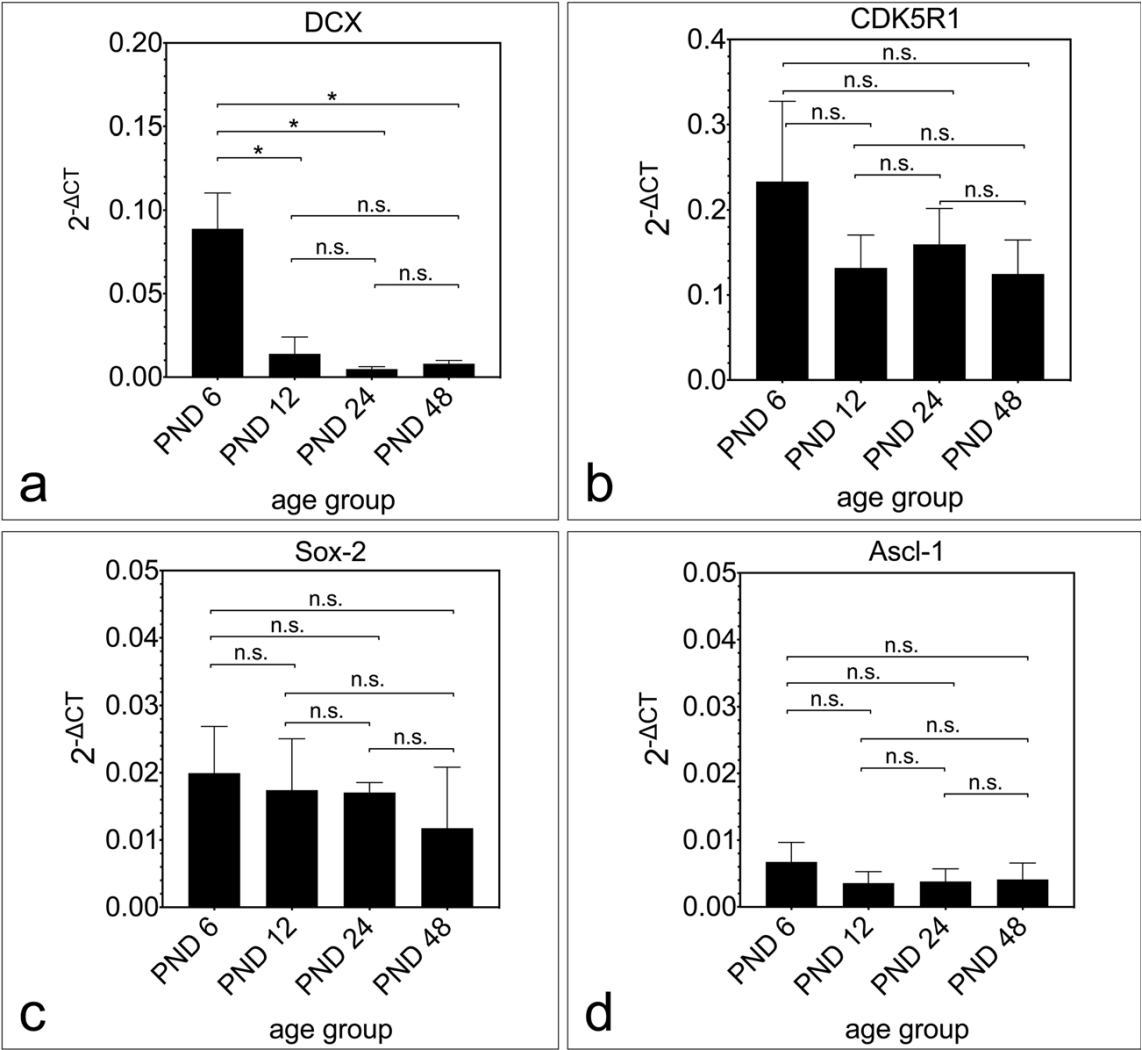
Expression of the genes of neural stem cell markers in rat IC from PND 6 to PND 48. **a–d** Representation of the age-dependent expression of the genes DCX, Sox-2, CDK5R1, and Ascl-1 using real-time quantitative PCR. **a** The neuronal migration protein DCX shows a significant decrease between PND 6 and PND 48 with the most significant decrease between PND 6 and PND 12. **b–d** The gene expression of the transcription factor Sox-2, the nestin phosphorylator CDK5R1, and the bHLH transcription factor Ascl-1 shows no significant changes between the age groups PND 6 to PND 48. The central horizontal bars show the mean; error bars depict the standard error of the mean; asterisks indicate the level of significance, *p < 0.05, **p < 0.005, ***p < 0.001, ****p < 0.0001

The expression of the genes DCX, CDK5R1, Sox-2, and Ascl-1 was studied. An expression of the investigated genes was found in all age groups (Fig. 8). The gene expression of DCX shows a significant decrease between PND 6 and the other age groups (PND 6 vs. PND 12, *p* = 0.0338; PND 6 vs. PND 24, *p* = 0.0172; PND 6 vs. PND 48, *p* = 0.0197). From PND 12 on, there were no significant differences in the gene expression of DCX with increasing age (PND 12 vs. PND 24, *p* = 0.4189; PND 12 vs. PND 48, *p* = 0.5937; PND 24 vs. PND 48, *p* = 0.2590) (Fig. 8a).

The gene expression of CDK5R1 (PND 6 vs. PND 12, *p* = 0.3745; PND 6 vs. PND 24, *p* = 0.5133; PND 6 vs. PND 48, *p* = 0.3473; PND 12 vs. PND 24, *p* = 0.6536; PND 12 vs. PND 48, *p* = 0.9032; PND 24 vs. PND 48, *p* = 0.5806) (Fig. 8b), Sox-2 (PND 6 vs. PND 12, *p* = 0.8180; PND 6 vs. PND 24, *p* = 0.7046; PND 6 vs. PND 48, *p* = 0.5130, PND 12 vs. PND 24, *p* = 0.9658; PND 12 vs. PND 48, *p* = 0.6584; PND 24 vs. PND 48, *p* = 0.5952) (Fig. 8c), and Ascl-1 (PND 6 vs. PND 12, *p* = 0.4023; PND 6 vs. PND 48, *p* = 0.5287; PND 12 vs. PND 24, *p* = 0.9287; PND 12 vs. PND 48, *p* = 0.8681; PND 24 vs. PND 48, *p* = 0.9303) (Fig. 8d) showed no significant differences with increasing age.

## Discussion

Previous studies have shown that there is strong evidence for the existence of NSCs in the rat neonatal IC [14, 15]. Since distinct markers of NSCs do not exist, this study aimed to analyze the neurogenic potential of the rat IC using indirect methods [7, 23]. The presented results indicate that neurogenetic potential is present in the rat IC into adulthood.

The tests were carried out on PND 6, 12, 24, and 48 rats in order to take into account all the critical stages of development of the rat IC. The age groups PND 6 and 12 represent the critical phase in the development of the rat’s auditory pathway. The rat’s hearing onset takes place around the 14th postnatal day. [24–26]. With 24 and 48 days, rats are either approaching sexual maturity or are at a stage shortly after sexual maturity, which is about 40 days [25] and can, therefore, be called adult. Thus, taking into account the ethical necessity of reducing the number of animals used in experiments, an attempt was made to choose intervals that are representative of essential stages of development, on the one hand, and allow a statement to be made about proportionality, on the other.

### Neurosphere-Forming Capacity

The ability of NSCs to mitotic self-renew has been demonstrated in cell culture studies, which are often described for this purpose in the literature. Cell cultures provide an in vitro analysis of the proliferation and self-renewal of NSCs for the evaluation of the neurogenetic potential [4, 7]. Primary, secondary, tertiary, and quaternary neurospheres formed in all age cohorts studied. The neurospheres of the primary cultures were analyzed in relation to the number of plated cells to achieve better comparability with similar studies. An age-related decrease in the number of neurospheres formed per 100,000 cells (PND 6 vs. PND 48, *p* < 0.001) (Fig. 1) was observed. The IC of 48-day-old rats showed a formation of about 348 neurospheres per 100,000 cells. Only a few comparable cell culture studies of similar brain regions are described in the literature. In one study, the formation of 68 neurospheres per 100,000 cells in the inner ear of the adult rat was described [8]. The CN of the adult rat showed a formation of 1 neurosphere per 100,000 cells [27]. In the dorsal vagal complex of adult rats, 760 neurospheres were formed per 100,000 cells [28].

Subsequently, the number of neurospheres per animal was plotted to show the proliferation potency within an age group along the passages. The number of neurospheres increased within all age groups with increasing passage (Fig. 2). In the literature, both passage studies of NSCs with increasing and decreasing number of neurospheres have been described [27, 28]. In one study an increasing number of neurospheres with an increased passage in cell cultures of the CN of neonatal mice was shown [13]. Following the results, the evaluation of the number of living cells in the cell cultures was depicted.

It is challenging to compare the results with those of the different studies because of the different approaches. The cell culture medium (DMEM vs. Neurobasal medium®), the way the passage was performed (enzymatically vs. mechanically; after 5 days vs. after 30 days), and the species studied (rat vs. mouse) are essential factors influencing the outcome of the studies. In previous studies, a different potency to form neurospheres between the CN and the IC of neonatal animals was shown [15]. Furthermore, the use of different growth factors and their concentration influences the formation and development of neurospheres [29]. However, there are studies of other brain regions that are methodologically comparable with this study. In a similar study of the forebrain, 500 primary neurospheres in PND 1 and about 150 primary neurospheres in PND 28 per 10,000 cells were found. This study also shows the relationship between age and the rate of neurosphere growth. With increasing age, a significantly longer time is required for neurosphere formation to occur [30]. Another comparable study showed a growth of about 35 primary neurospheres per 10,000 cells from cells of the hypothalamus (including the 3rd ventricle) of the rat under similar preparation and culture conditions [31]. Considering that the hypothalamus is a brain region that has been extensively examined for neural stem cells and is neurogenically active, the potency of the inferior colliculus to form primary neurospheres is comparable.

The passage studies show that the isolated cells of the IC of all age groups examined theoretically have the ability to renew themselves without limitation in vitro. An age-dependent decrease in this proliferative potency was observed between the age groups. This quality is one of the main criteria of NSCs and can be demonstrated using the neurosphere assay [32].

### Stem Cell Proliferation

In previous studies, NSC markers were immunocytologically detected in neurospheres generated from the IC of 6-day-old rats [15]. Thus, the question arose as to whether different NSC markers were detectable in the neurospheres of the IC until adulthood. Nestin is an intermediate filament that is expressed in the early stages of neuroepithelial cell development and is detectable until the terminal differentiation of the cells [33]. Furthermore, the NSC markers Sox-2, Musashi-1, and DCX were investigated. Sox-2 is an HMG-box transcription factor detectable in multipotent NSCs of all developmental stages [34]. Musashi-1 is an RNA-binding protein that is highly enriched in Nestin-positive neuroepithelial cells and whose expression is no longer detectable after neuronal differentiation [35]. The basic helix-loop-helix (bHLH) transcription factor Atoh1 plays a decisive role in the development of the central auditory pathway and has been detected in neurospheres, as well as in its branches [12, 27, 36, 37]. Moreover, the neurospheres were examined for DCX, a protein that is important for migrating and differentiating neurons [38]. The expression of all examined NSC markers was immunocytologically demonstrated in the neurospheres of PND 6 to PND 48 rats (Fig. 4). The NSC markers were shown not only in the cells of the neurospheres but also in the emigrating cells (Fig. 4). These cells may be attached neural progenitor cells with an ability to grow on a monolayer [39]. Both cells within the neurospheres and cells emigrating from them showed Nestin-positive staining (Fig. 4). The detection of Sox-2- and Atoh1-positive cells was limited to the nucleus and took place in cells within the neurospheres as well as in the emigrating cells (Fig. 4). These observations are consistent with comparable studies [27, 34]. As shown in corresponding studies, localized Musashi-1 areas in the cytoplasm and the nuclei were found to be positively stained (Fig. 4) [35, 40]. DCX positively stained cells were localized in the cytoskeleton of the cells (Fig. 4). In the literature, it was shown that DCX is associated with the proteins of the microtubules and that co-labeling with Nestin is typical for progenitor cells with neuronal determination [38, 41].

NSC marker–positive cells were compared with DAPI-positive cells to show the expression of NSC markers in the neurospheres of all age groups studied over time. The investigation of the NSC markers Sox-2 and Atoh1 showed a decrease of the expression with increasing age (Fig. 5). In the literature, a decrease of Sox-2-positive cells in the subventricular zone has been described [42]. The expression of Atoh-1 in the cochlear nucleus decreased significantly with increasing age [27].

These results show that the neurospheres developed from the isolated cells of the IC in all age groups studied contain cells that are positive for different stem cell and progenitor markers, and that the expression of these markers follows age-related patterns.

### Capacity of Differentiation

Another characteristic of NSCs is their ability to differentiate into all cells of the neuroectodermal cell line [39]. Specific markers were used to analyze the differentiation ability of the isolated single cells. β-III-tubulin is a protein of the microtubules and part of the tubulin family, which is formed almost exclusively by neurons in the early stages of development [43]. β-III-tubulin-positive cells whose cell bodies and axons were stained positively were found (Fig. 6a). MBP identifies the myelination processes of oligodendrocytes [44]. The distal extensions of cells with prominent soma were stained MBP-positive, allowing them to be identified as mature oligodendrocytes (Fig. 6b). Mature astrocytes were identified by GFAP, an astrocyte-specific intermediate filament (Fig. 6c) [45]. Nestin positively stained cells were found whose morphology and immunocytological staining were compatible with the characteristics of neural progenitor cells (NPC) (Fig. 6d) [33]. The identification of progenitor cells, neurons, oligodendrocytes, and astrocytes were shown in the single cells derived from the neurospheres of PND 6 to PND 48 animals. The marker-positive single cells were related to all β-tubulin-positive cells to demonstrate the capacity of differentiation. The evaluation of all β-III-tubulin(+), MBP(+), and Nestin(+) ratios showed a significant decrease in the respective cell numbers between early postnatal (PND 6) and adult animals (PND 48) (Fig.6e, g, h). In literature, an age-dependent decrease of MBP in the auditory nerve of mice [46], a 7-fold higher level of β-III-tubulin mRNA in corticospinal neurons in 8-day-old than in adult hamsters [47], and a decrease in Nestin expression in the CN of adult rats were described [27]. In GFAP, however, an increase in the GFAP(+)/β-tubulin(+)-ratio was observed with increasing age (Fig. 6f), which was also described in the CN of rats in the first year of life and other sections of the CNS [48, 49]. The reasons for this increase seem to be multifactorial and could be a trophic change due to decreasing afferent stimulation or age-related degenerative changes in neuronal structures [49].

These results show that the neurospheres, formed in free-floating cell cultures, have the ability to differentiate into all cell types of the neuroectodermal cell line in all age groups studied. However, the neuroectodermal cell lines showed a different and characteristic capacity for differentiation over time.

### NSC Markers in Histological Sections

Since the markers Sox-2, Nestin, Musashi-1, and Atoh1 were detected in neurospheres generated from the IC, the question arose whether these stem cell markers can also be identified in histological sections of the IC. The stem cell marker Atoh1 showed co-staining with DAPI (Fig. 7m–p). This staining behavior of Atoh1 is known from previous studies [50]. Musashi-1 showed a predominant staining of the perikarya [40] (Fig. 7i–l). In the nuclei, co-labeling of Sox-2 and DAPI was observed (Fig. 7 e-h). Sox-2 labeling is reported to be specific to the nucleus [51]. In the histological sections, stained with β-III-tubulin and Nestin, no co-labeling of both markers was detectible, which indicates that the Nestin-positive cells are undifferentiated neural precursor or stem cells. Nestin was stained in cytoplasmatic structures and, therefore, not in the cell nuclei (Fig. 7a–d).

Therefore, the detection of NSC markers up to the adult stage was achieved not only in neurospheres from in vitro cultures of the IC, but also in histological sections. This finding supports the assumption of a neurogenic niche in the rat IC until adulthood.

### Molecular Genetic Analysis of NSC Markers

NSC markers were detected in neurospheres and histological sections of the IC. The question arose whether NSC markers and factors that have a decisive influence on neurogenesis can be detected at the molecular genetic level. The gene expression of DCX, a marker for migration and differentiation of neurons, peaked at PND 6 with a significant decrease compared with older age groups (Fig. 8a). DCX is a specific marker for neurogenesis in adult neural tissue, and a reduction in the expression of DCX with increasing age is known from previous studies [52, 53]. The organization and regulation of the intermediate filament Nestin at the molecular genetic level is significantly influenced by CDK5R1 (p35). This activates Nestin-associated CDK5 and plays a vital role in postmitotic neuronal differentiation [54–56]. The gene expression of CDK5R1 shows no significant changes over time (Fig. 8b). The expression of mRNA of CDK5R1 was shown in adult rat IC by other studies [57]. The gene expression of the HMG-box transcription factor Sox-2 showed a stable expression level and no significant changes with increasing age (Fig. 8c). This is in contrast to the relative decrease in the neurospheres observed in this study and the decline in the number of Sox-2-positive cells in the subventricular zone described in the literature. The same study also describes a twofold increase in relative gene expression of Sox-2 between embryonic and 3 to 5 months old animals [42]. In the IC of the rat, no increase in gene expression was found in this study, but a constant gene expression level of Sox-2 in a comparable period. The divergence between the molecular genetic results and the immunocytological results suggests a crucial role in the posttranscriptional and posttranslational modification of Sox-2 [58]. The proneural gene Ascl-1 is a transcription factor of the basic helix-loop-helix class. It plays an essential role in the maturation of NSC and in the development of progenitor cells into differentiated neurons [59]. In addition to the basic helix-loop-helix transcription factor Atoh1 detected in the neurospheres, and histological sections, the factor Ascl-1 from the same class was detected at the molecular genetic level, which plays an important role in the maturation of neurons in the IC [60]. Gene expression of Ascl-1 was detectable in all age groups studied and showed no significant changes over time (Fig. 8 d). Ascl-1 has been identified in the adult stage as a marker of long-term neurogenetic cell populations [61].

These results show that NSC markers in IC are detectable to the adult stage at the molecular genetic level.

## Conclusion

Up to adult age, the ability of mitotic self-renewal and proliferation and the ability to differentiate into neural progenitor cells and all cell types of the neuroectodermal cell lines were demonstrated in cells isolated from the rat IC. In summary, these results indicate the existence of a persistent stem cell niche in the IC rats until adulthood.

However, there is an age-related decline in this potential. In early postnatal animals, passage evaluations showed that their proliferation potency is significantly more pronounced than in adult stages. This is also reflected in the differentiation capacity results, which showed age characteristic features and indicated that progenitor cells in adult animals have a lower capacity to differentiate into all cell types of the neuroectodermal lineage. The evaluation of NSC markers in the neurospheres and at the molecular genetic level describes the same tendency. Interestingly, acoustic deprivation of rats younger than 14 days leads to diffuse immature patterns of innervation between the cochlea and the IC [62]. The hearing onset of rats also takes place at about this point in time [63]. It can be assumed that neurogenesis in young animals has a decisive influence on the development of the auditory pathway. Furthermore, a variety of neurogenetic moderators were discovered, which may allow a specific influence on neurogenesis in the future [8]. The genetic modification of neural stem cells in vitro and in vivo offers increasing possibilities to influence individual genetic factors [64]. Likewise, the epigenetic control of neural stem cells requires the identification of factors that play an essential role in the proliferation, maturation, and differentiation of neural stem cells [65]. If it were possible to influence neurogenesis specifically, this would open up new therapeutic possibilities for the treatment of neurodegenerative or traumatic diseases. However, this requires a further and more profound knowledge of the cellular and molecular genetic processes of neurogenesis.

## Data Availability

The data used to support the findings of this study are included within the article.
